# A role of salt bridges in mediating drug potency: A lesson from the N-myristoyltransferase inhibitors

**DOI:** 10.3389/fmolb.2022.1066029

**Published:** 2023-01-10

**Authors:** Danislav S. Spassov, Mariyana Atanasova, Irini Doytchinova

**Affiliations:** Department of Chemistry, Faculty of Pharmacy, Medical University of Sofia, Sofia, Bulgaria

**Keywords:** salt bridge, ligand-protein complex, drug-protein interactions, NMT, protein conformation and drug potency, N-myristoltransferase, conformational stabilization and inhibition, salt bridge and protein conformation

## Abstract

The salt bridge is the strongest non-covalent interaction in nature and is known to participate in protein folding, protein-protein interactions, and molecular recognition. However, the role of salt bridges in the context of drug design has remained not well understood. Here, we report that a common feature in the mechanism of inhibition of the N-myristoyltransferases (NMT), promising targets for the treatment of protozoan infections and cancer, is the formation of a salt bridge between a positively charged chemical group of the small molecule and the negatively charged C-terminus of the enzyme. Substituting the inhibitor positively charged amine group with a neutral methylene group prevents the formation of the salt bridge and leads to a dramatic activity loss. Molecular dynamics simulations have revealed that salt bridges stabilize the NMT-ligand complexes by functioning as molecular clips that stabilize the conformation of the protein structure. As such, the creation of salt bridges between the ligands and their protein targets may find an application as a valuable tool in rational drug design.

## 1 Introduction

The salt bridge is a non-covalent interaction that combines an electrostatic attraction between oppositely charged chemical groups or atoms and a hydrogen bond; hence, its strength exceeds the strength of a simple hydrogen bond (Donald et al., 2011; Ferreira de Freitas and Schapira, 2017). In proteins, the salt bridges occur most frequently between the positively charged basic amino acid residues Lys or Arg and the negatively charged acidic Asp or Glu residues (Kumar and Nussinov, 2002; Bosshard et al., 2004; Donald et al., 2011; Basu and Mukharjee, 2017; Ferreira de Freitas and Schapira, 2017). In this context, salt bridges are known to participate in protein-protein interaction, protein folding, protein recognition, protein conformational rigidity, and protein stability (Takano et al., 2000; Kumar and Nussinov, 2002; Bosshard et al., 2004; Basu and Mukharjee, 2017; Ferreira de Freitas and Schapira, 2017). However, much less is known about salt bridges and their significance in mediating the interaction between small molecule ligands and their protein targets. A substantial number of drug molecules contain either positively or negatively charged chemical groups and, as such, might be capable of participating in salt bridges within their binding sites (Uddin et al., 2021). Indeed, systematic surveys of the protein-ligand complexes deposited in the protein databank have identified over a thousand unique small molecule ligands that form salt bridges with their protein targets (Ferreira de Freitas and Schapira, 2017; Kurczab et al., 2018). Such prevalence raises questions about the significance of salt bridges in drug-protein interactions and their role in mediating inhibitor potency.

Hitherto, little is known about the role of these salt bridges in protein-ligand complexes. In several cases, such as for certain inhibitors of the Epidermal growth factor receptor (EGFR) (Peng et al., 2013) and G-protein coupled receptors ligands (Ferreira de Freitas and Schapira, 2017), it has been shown that the salt bridge plays a crucial role in the ligand’s activity. However, in the prevailing number of cases, the salt bridges, their role in protein-ligand interactions, their physical and chemical properties, and the mechanisms that mediate their effects on the potency of the compounds have remained largely unexplored. The incomplete understanding of the role of salt bridges in protein-ligand interactions represents an obstacle to drug design, as the researchers are unaware of their significance and how to use them to boost the inhibitors’ potency.

This study began with the observation that two structurally unrelated inhibitors of N-myristoyltransferases (NMT)—IMP-1088 and DDD85646 that were developed independently of each other by high-throughput screening and fragment-based approaches form a salt bridge within their binding sites. IMP-1088 and DDD85646 are very potent NMT inhibitors with reported IC_50_ in the picomolar and low nanomolar range, respectively (Frearson et al., 2010; Mousnier et al., 2018). In preclinical models, the NMT inhibitors were found to be very effective for treating parasitic protozoan infections such as the African sleeping sickness and are promising therapeutics for the treatment of other protozoan diseases such as malaria and leishmaniasis (Frearson et al., 2010; Ritzefeld et al., 2018; Schlott et al., 2018). In addition, NMT inhibitors display potent anti-tumor activity and have produced complete anti-tumor responses in preclinical murine models (Thinon et al., 2016; Beauchamp et al., 2020). NMTs are enzymes that catalyze the myristoylation of selected cellular proteins, which contain a specific peptide sequence known as a myristoylation signal (Thinon et al., 2014). This sequence is located in the N-terminal region of the proteins and binds into a specially evolved pocket in the NMT active site, known as the peptide binding pocket (Thinon et al., 2014; Dian et al., 2020). NMT also uses a cofactor—Myristoyl-CoA (Myr-CoA), which binds to a site adjacent to the peptide binding pocket (Thinon et al., 2014; Dian et al., 2020). During myristoylation, the myristic acid is transferred from Myr-CoA to the N-terminus of the myristoylated proteins (Thinon et al., 2014; Dian et al., 2020). The myristic acid, due to its hydrophobicity, is used for the attachment of the modified proteins to the cellular membranes but also facilitates the dimerization of interacting partners and regulates crucial cell signaling events (Patwardhan and Resh, 2010; Gaffarogullari et al., 2011; Spassov et al., 2018; Meinnel et al., 2020).

NMTs are an ancient enzyme family represented in human by two members—NMT1 and NMT2, which share a highly conserved catalytic domain (Ducker et al., 2005; Meinnel et al., 2020). IMP-1088 and DDD85646 are not selective against the two forms and inhibit them with almost identical potency (Frearson et al., 2010; Mousnier et al., 2018).

**FIGURE 1:**
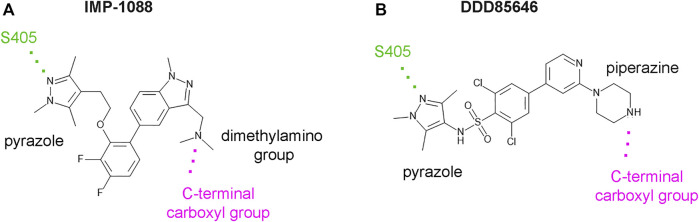
IMP-1088 and DDD85646 share a common mode of interaction in the active site of NMT. **(A)**. The main polar interactions between IMP-1088 and *Homo sapiens* NMT1 based on the crystallographic structure PDB 5MU6. **(B)**. The main polar interactions between DDD85646 and *Homo sapiens* NMT1 based on the crystal structure PDB 3IWE. Both inhibitors have a pyrazole ring that forms a hydrogen bond with S405 (shown in green). IMP-1088 and DDD85646 also interact with the C-terminal carboxyl group of NMT protein (shown in magenta) through a dimethylamino group and a piperazine ring, respectively.

**FIGURE 2:**
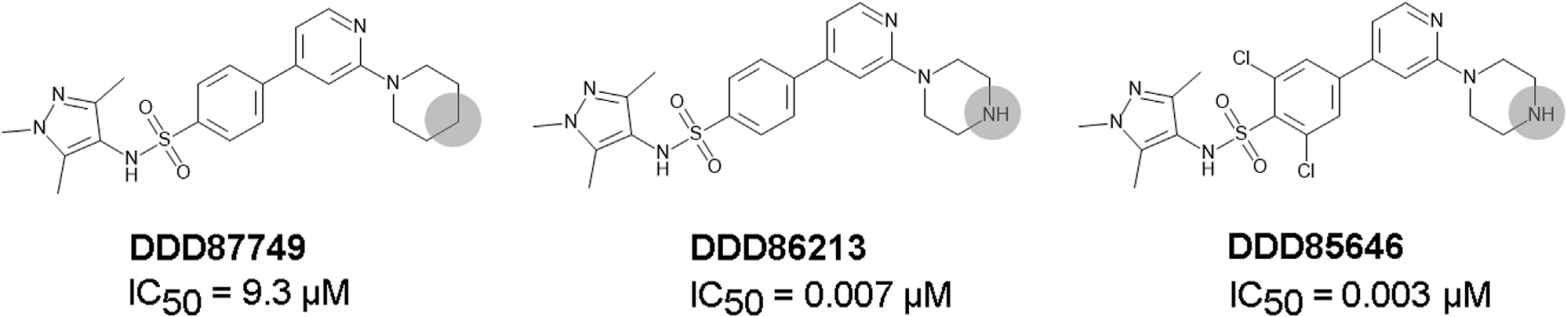
The role of the nitrogen atom of the piperazine ring in the potency of the DDD85646 series of compounds. IC_50_ towards *Homo sapiens* NMT1 is shown. Substitution of the indicated nitrogen atom with a carbon reduces potency by 1,328-fold.

DDD85646 and IMP-1088 share a common mode of interaction with NMT (Figure 1). Both compounds contain a pyrazole ring that participates in a hydrogen bond with Ser405 in the catalytic center of NMT. In addition, the crystallographic structures of NMT complexes with these inhibitors have revealed an interaction between the C-terminal carboxyl group of NMT and the piperazine ring of DDD85646 or the dimethylamino group of IMP-1088, both of which contain a terminal basic nitrogen atom (Figure 1). Evidence indicates that in the DDD85646 series, the terminal basic nitrogen is crucial for activity. Specifically, this can be seen in examples of compounds where the nitrogen atom is substituted for a carbon atom (Figure 2). For example, a comparison between DDD86213 and DDD87749, two compounds intermediary in the development of DDD85646, reveals that substituting the nitrogen atom of the piperazine ring reduces the inhibitory potency of the ligand by 1,328-fold (from IC_50_ 7 nM to IC_50_ 9.3 µM) (Figure 2) (Brand et al., 2017). In the case of the IMP-1088 series, intermediary compounds at which the nitrogen atom of the dimethylamino group is replaced with carbon have not been tested experimentally because the fragment that contains the dimethylamino group was identified by high throughput screening and was present in the initial hit of the series—IMP-72 (Mousnier et al., 2018). Irrespective of its crucial role in the activity of the NMT inhibitors, the interaction with the C-terminus of NMT has not been studied in detail, and the many aspects of this interaction have not been described previously. Here we report for the first time that the NMT inhibitors are positively charged in the physiological environment due to the protonation of the critical basic nitrogen atom and that the positively charged chemical groups of the inhibitors participate in a salt bridge with the negatively charged carboxyl group at the C-terminus of the NMT protein. Using Molecular dynamics (MD) simulations of protonated and non-protonated forms of the inhibitors, we demonstrate that the salt bridge has an unexpected role in stabilizing the NMT protein conformation and that this may be a significant factor in mediating its effects on NMT inhibitors’ potency.

## 2 Materials and methods

### 2.1 Visualization of protein-ligand interactions and image preparation

Protein-ligand interactions were visualized in PyMOL 1.6.0.0 (Schrödinger, New York, United States) (Schrödinger and DeLano, 2020) and YASARA v. 20.4.24 (IMBM, University of Graz, Austria) (Krieger and Vriend, 2014). The protonation state of the NMT inhibitors inside their complexes with N-myristoyltransferases was determined by YASARA and Protoss (University of Hamburg, Germany) (Bietz et al., 2014). pKa determinations were performed using Epic software from the Maestro package release 2018-4 (Schrödinger, New York, United States) and rely on extensions to the well-established Hammett and Taft approaches for pKa prediction, namely, mesomer standardization, charge cancellation, and charge spreading to make the predicted results reflect the nature of the molecule itself rather just for the particular chemical group (Shelley et al., 2007; Schrödinger and DeLano, 2020. The partial charges of the molecules were determined by using the Maestro package release 2018-4 (Schrödinger, New York, United States). Crystal structure images and superimpositions were prepared in PyMOL 1.6.0.0 (Schrödinger, New York, United States). The distances and angles of the salt bridge were determined in PyMOL based on the crystal structures of IMP-1088 and DDD85646 complexes with human NMT1 (PDB 5MU6 and PDB 3IWE, respectively). The 2D structures of the NMT inhibitors were created in MedChem Designer v.5.5 (SimulationsPlus, Lancaster, CA, United States). The nomenclature of the secondary structural elements in NMT, such as the names and the position of the α–helices, β-sheets, or connecting loops, was adopted from Dian et al. (Dian et al., 2020).

### 2.2 Molecular dynamics simulations

The protonated and unprotonated forms of IMP-1088 and DDD8646 were prepared using the crystal structures of their complexes with *Homo sapiens* NMT1 and Myr-CoA (PDB 5MU6 and 3IWE, respectively). The hydrogen atoms were added to the inhibitors’ structures in YASARA. YASARA automatically assigns the protonation and charge of the ligands at pH 7.4 to generate the protonated charged forms of the NMT inhibitors. The unprotonated uncharged forms of the NMT inhibitors were generated after the removal of the hydrogen atom, participating in the salt bridge. The structure of DDD86213 was prepared by replacing the chlorine atoms from DDD85646 with hydrogen atoms and of DDD87749 by replacing the nitrogen donor atom in DDD86213 with a carbon atom using PyMOL. The NMT inhibitor structures were saved in mol2 format and prepared for MD simulations using the Antechamber program from the AMBER v. 18 package (UCSF, San Francisco, United States) (Case et al., 2005; Case et al., 2018) by assigning a net charge of 0 for the non-protonated forms and net charge +1, for the protonated forms. Myr-CoA was similarly prepared for MD simulations using the Antechamber program from the AMBER v. 18 package. The NMT protein used for MD simulations contained an N-terminal ACE cap and no C-terminal cap. The addition of a C-terminal cap was not possible because it could have interfered with the interaction with the ligand’s positively charged group and was not necessary because the C-terminus of the native NMT protein contains a negatively charged carboxyl group at this position. The crystal structures of NMT- IMP-1088/DDD85646 complexes do not contain the full-length NMT protein and have truncated N-terminal parts (the first 114 amino acids). Thus the addition of the N-terminal ACE cap is necessary to eliminate the positively charged N-terminus at position 115 that is not normally present in the full-length protein. The ternary complexes, consisting of NMT protein, Myr-CoA, and either the protonated or the unprotonated forms of IMP-1088, DDD85646, DDD86231, or DDD87749 were solvated in saline (0.9% sodium chloride) in a truncated octahedral box, containing 15,891 water molecules (distance between protein and the edge of the box varied between 23.7–39.7 Å), energy minimized, heated to 310 K at constant volume for 1 ns, density equilibrated at 1 bar for 1 ns, equilibrated keeping constant T and *p* for 1 ns, using the Langevin thermostat (Adelman and Doll, 1974) and Berendsen barostat (Berendsen et al., 1984) and simulated for 1,000 ns by AMBER v. 18 (UCSF, San Francisco, United States) (Case et al., 2005; Case et al., 2018). During all steps of simulations, i.e., heating, density equilibration, preproduction, and production dynamic SHAKE algorithm (Ciccotti and Ryckaert, 1986) was used for constraining covalent bonds involving hydrogen with a 2 fs time step. The bonds to hydrogen were not constrained only during the energy minimization step. The systems were simulated with the ff14SB force field (Maier et al., 2015) under periodic boundary conditions. Frames were saved every 1 ns to generate 1,000 frames for a total of 1 µs duration of MD production simulations.

### 2.3 Analysis of results from MD simulations

The data from the MD simulations were analyzed in VMD (Visual Molecular Dynamics, the University of Illinois at Urbana–Champaign, United States) (Humphrey et al., 1996). For determining the distance between the nitrogen atom of the ligand and the oxygen atom at the C-terminus of the NMT protein, the atoms were initially selected through the Graphic and Labels option in VMD, and the exported graphical data was used to generate the charts in GraphPad Prism.

RMSD (root-mean-square deviations) of the inhibitors, NMT protein, and Myr-CoA were determined by the RMSD trajectory tool in VMD. The NMT protein corresponds to resid 1 to 382 (includes all 382 amino acid residues present in the crystal structure PDB 3IWE), the A' –helix- A’a–loop region to resid 1 to 19, the NMT inhibitors to resid 383, and the Myr-CoA to resid 384. In all cases, the RMSD values were determined for the heavy atoms, e.g., excluding the hydrogen atoms. The exported RMSD graphical data was used to generate the graphs in GraphPad Prism.

## 3 Results

### 3.1 DDD85646 and IMP-1088 are positively charged at pH 7.4

**FIGURE 3:**
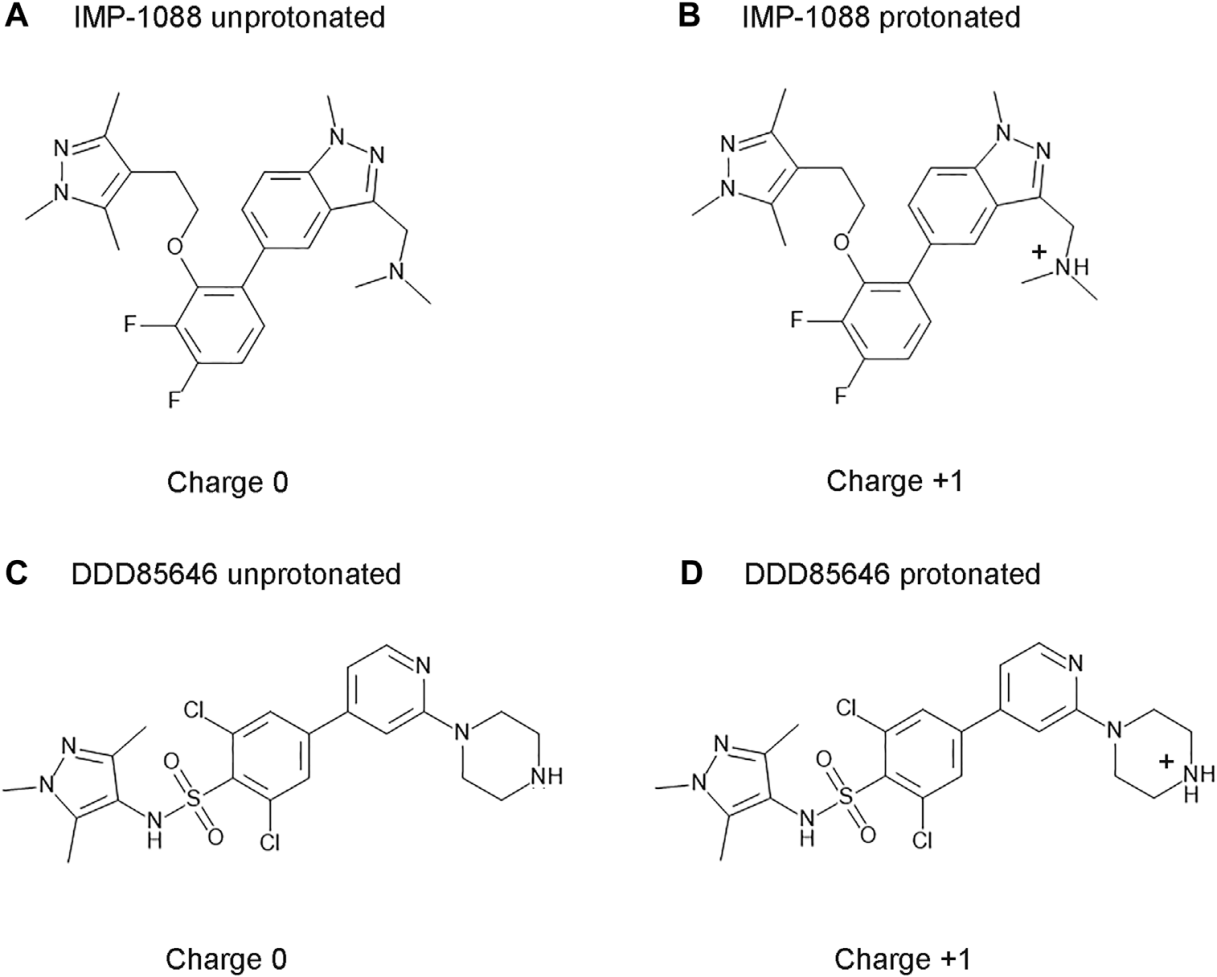
Structures and protonation forms of the NMT inhibitors IMP-1088 and DDD85646. **(A)** Unprotonated IMP-1088. **(B)** Protonated IMP-1088. **(C)** Unprotonated DDD85646. **(D)** Protonated DDD85646. IMP-1088 contains a dimethylamino group, and DDD85646 has a piperazine ring, and their protonation gives the molecules a positive +1 charge. At pH 7.4, IMP-1088 and DDD85646 are predicted to be in their protonated, positively charged forms.

IMP-1088 contains a dimethylamino group, and DDD85646 has a piperazine ring that can become protonated. As such, the compounds can exist in two forms—an unprotonated uncharged and a protonated charged form (Figure 3). The targets of these inhibitors - NMT1 and NMT2, are intracellular cytoplasmic proteins that exist in an environment where the pH has been estimated to be 7.0-7.4 (Flinck et al., 2018; Persi et al., 2018). The pKa values for trimethylamine and piperazine, determined experimentally, are 9.8 and 9.73, respectively (Khalili et al., 2009; Settimo et al., 2014), indicating that these basic groups exist almost entirely in a cation form in the physiological environment. In the context of the whole structure of the inhibitors, the predicted pKa for the dimethylamino group of IMP-1088 and the piperazine of DDD85646 by using the Epic package of Maestro (Schrodinger) was 8.39 and 8.78, respectively. Hence, in the physiological environment, most IMP-1088 and DDD85646 molecules are expected to be charged due to the protonation of their terminal nitrogen atom (Figure 3). The protonation of IMP-1088 and DDD85646 was also confirmed in YASARA (Krieger and Vriend, 2014) and Protoss (Bietz et al., 2014), and they both indicated the presence of the protonated forms of the inhibitors inside the binding sites of their complexes with NMT. DDD86213 has a predicted pKa value of 8.78, identical to DDD85646. In contrast, DDD87749 is not protonated and not charged due to the substitution of the terminal nitrogen atom with a carbon - the pKa for this compound lies outside the normal range of sampled pH range in Maestro. Therefore, DDD87749 models the interaction between the non-protonated inhibitors and NMT under physiological pH conditions.

### 3.2 IMP-1088 and DDD85646 form a salt bridge with the C-terminus of the NMT protein

**FIGURE 4:**
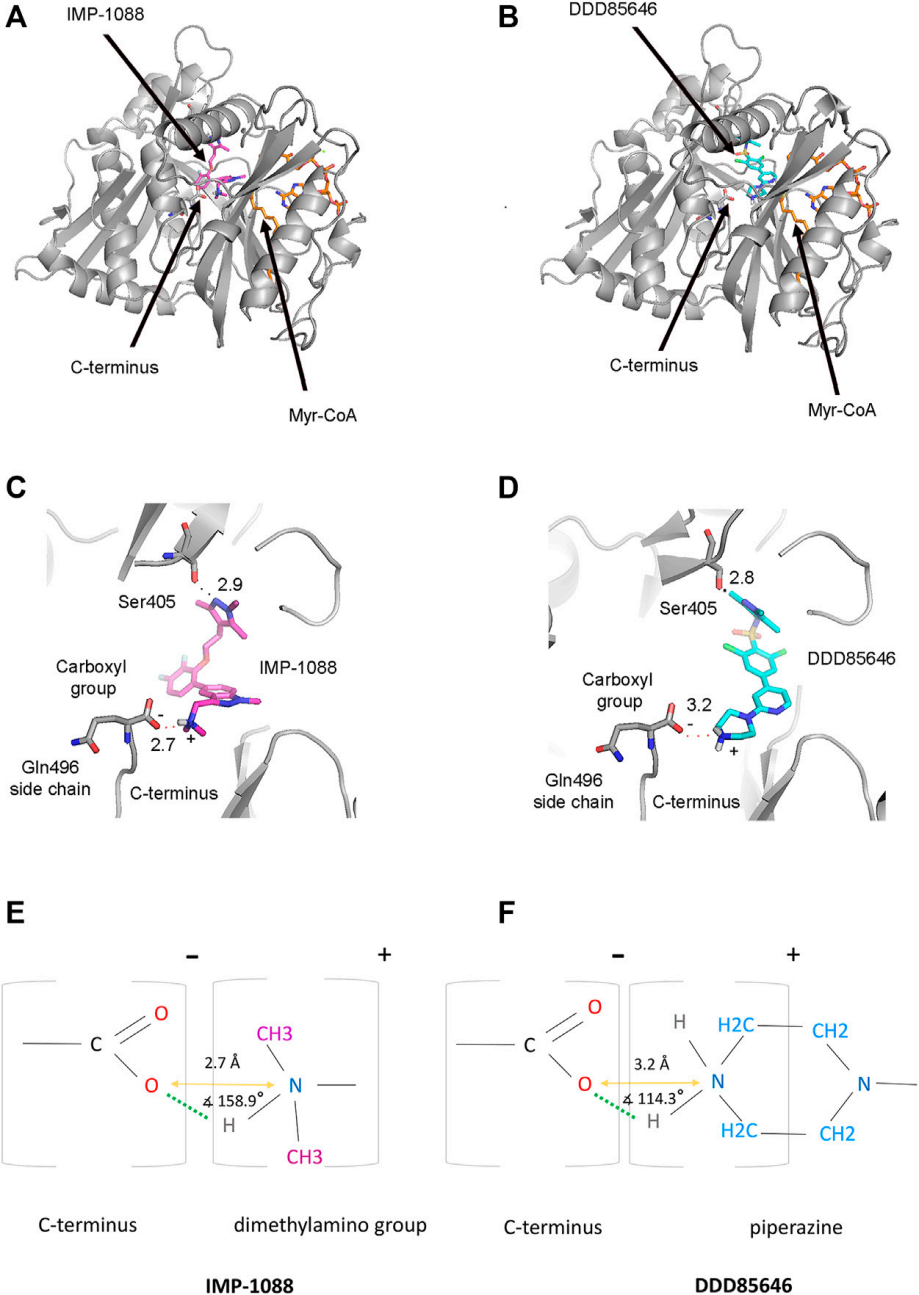
Formation of a salt bridge between the NMT inhibitors and the C-terminus of the NMT protein. **(A)** The crystal structure of *Homo sapiens* NMT1 in complex with IMP-1088 and Myr-CoA (PDB 5MU6). IMP-1088, shown in magenta, occupies the peptide-binding pocket of NMT and is located nearby the cofactor Myr-CoA, shown in orange. **(B)** The crystal structure of *Homo sapiens* NMT1 in complex with DDD85646 and Myr-CoA (PDB 3IWE). DDD85646 is shown in cyan and Myr-CoA in orange. **(C)** Formation of a salt bridge between IMP-1088 and the C-terminus of NMT in PDB 5MU6. The hydrogen bond with Ser405 is also indicated. **(D)** Formation of a salt bridge between DDD85646 and the C-terminus of NMT in PDB 3IWE. **(E)** A schematic representation of the salt bridge between the dimethylamino group of IMP-1088 and the C-terminal carboxyl group of NMT. **(F)** A schematic representation of the salt bridge between the amine group of the piperazine ring of DDD85646 and the carboxyl group at the C-terminus of NMT. The charge of the groups is indicated above the brackets. The length of the salt bridge, defined as the distance between the oxygen atom at the C-terminus of NMT and the nitrogen atom of the ligand (orange arrow), is shown in angstroms (Å). The angle between the ligand nitrogen atom, hydrogen atom, and the C-terminal carboxyl group oxygen atom is also indicated. Green dots indicate the hydrogen bond.

N-myristoyltransferases are enzymes that contain two binding pockets—one for binding the cofactor Myr-CoA and the other for the substrate peptide (Dian et al., 2020). The crystallographic structures of NMT in complex with the NMT inhibitors IMP-1088 (PDB 5MU6) and DDD85646 (PDB 3IWE) reveal that the NMT inhibitors occupy the peptide binding pocket of NMT, and the cofactor Myr-CoA binds to a distinct region proximal to the inhibitors’ binding site (Figures 4A,B) (Frearson et al., 2010; Mousnier et al., 2018). In this binding mode, the NMT inhibitors and Myr-CoA occupy nearby sites but do not interact directly with each other (Frearson et al., 2010; Mousnier et al., 2018). To illuminate the role of the positively charged group of the NMT inhibitors, we analyzed the crystallographic structures of complexes of IMP-1088 and DDD85646 with human NMT1. In these structures, it is evident that the positively charged chemical group of the inhibitor forms a salt bridge with the negatively charged C-terminus of the NMT protein, which is located in the active site of the enzyme (Figures 4C,D) (Dian et al., 2020). The salt bridge’s formation involves the free carboxyl group of the C-terminal amino acid residue - Gln496, and not its side chain, which is oriented in the opposite direction (Figures 4C,D). The average pKa value of the carboxyl group at the C-terminus of folded proteins is estimated to be 3.3 (Grimsley et al., 2009), indicating that in the cytoplasm of the cell (pH 7.0-7.4), the C-terminus is expected to be deprotonated, negatively charged, and therefore available for the formation of the salt bridge.

**FIGURE 5:**
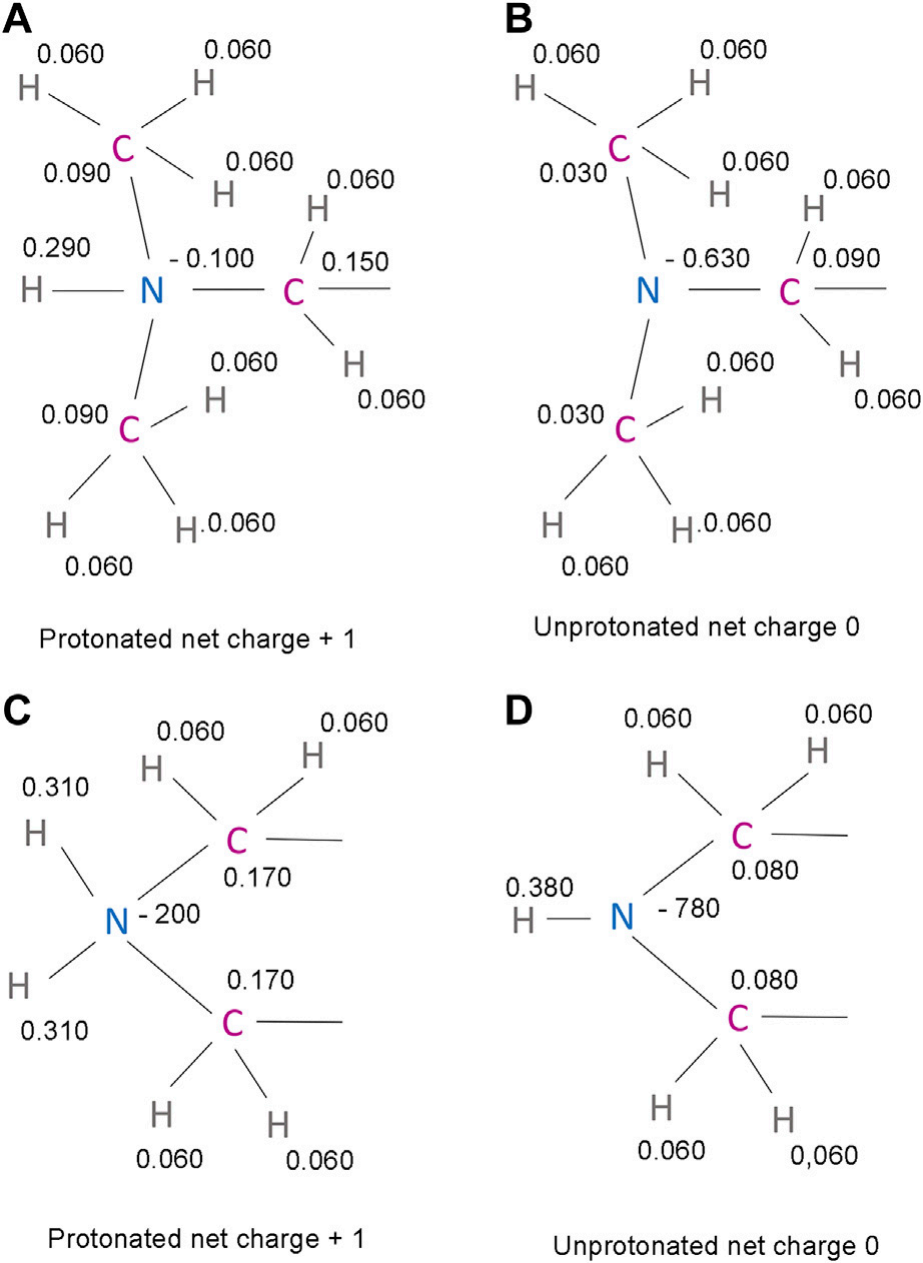
Predicted partial charges in the protonated and unprotonated forms of IMP-1088 and DDD85646 **(A)** The protonated dimethylamino group of IMP-1088. **(B)** The unprotonated dimethylamino group of IMP-1088. **(C)** The protonated amine group of DDD85646. A part of the piperazine ring is shown **(D)** The unprotonated amine group of DDD85646. A part of the piperazine ring is shown. Partial charges were determined using the Maestro software package release 2018-4 (Schrödinger, New York, United States of America) (Shelley et al., 2007; Schrödinger and DeLano, 2020).

Generally, a salt bridge combines two non-covalent interactions - an electrostatic attraction between chemical groups of opposite charges and a hydrogen bond (Donald et al., 2011). Both of these interactions can be identified in the complexes of NMT inhibitors. The electrostatic attraction involves the positively charged dimethylamino group of IMP-1088 or the amine group in the piperazine ring of DDD85646 and the negatively charged C-terminal carboxyl group of NMT (Figures 4E, F). A hydrogen bond between the protonated inhibitor’s hydrogen atom and the carboxyl group’s oxygen atom at the C-terminus of NMT is also formed, fulfilling the second requirement for forming a salt bridge (Figures 4E,F). The length of the salt bridge in the IMP-1088 and DDD85646 complexes is 2.7 and 3.2Å, respectively. (Figures 4E,F). Since the permissible distance for a salt bridge length is <4 Å, the measured distances are consistent with salt bridge formation between the inhibitors and NMT (Donald et al., 2011). Generally, the optimum angle between the nitrogen, hydrogen and oxygen atoms in the salt bridge is between 110° and 180° (Kurczab et al., 2018). The measured angles in the salt bridges of IMP-1088 and DDD85646 of 158.9° and 114.3° (Figures 4E,F) are in the range of values determined for different salt bridges in the crystallographic structures deposited in the protein databank (Kurczab et al., 2018). IMP-1088 may form a stronger salt bridge than DDD85646, considering that the length and the angle of the salt bridge in the complexes of this compound are more optimal for binding (Figures 4E,F). The distribution of partial charges of the protonated and unprotonated dimethylamino group and piperazine ring of the NMT inhibitors were determined by using the Maestro software (Schrödinger, New York, United States) (Shelley et al., 2007; Schrödinger and DeLano, 2020) (Figure 5). The nitrogen atom of these groups is electronegative, and the positive charge of the protonated form is distributed among the nearby hydrogen and carbon atoms (Figure 5). The highest partial positive charge is observed on the hydrogen atom participating in the salt bridge formation (Figure 5). The distribution of partial charges is shown in the absence of interactions, and the formation of a salt bridge between the inhibitors and the electronegative C-terminal carboxyl group may affect the described distribution.

The interaction between the inhibitors and NMT also involves forming a hydrogen bond with Ser405 (Figure 1), multiple π-π stacking, and hydrophobic interactions with several aromatic residues, including Tyr296, His298, Phe188, and Phe311.

### 3.3 The salt bridge contributes to the stability of the NMT-ligand complexes

**FIGURE 6:**
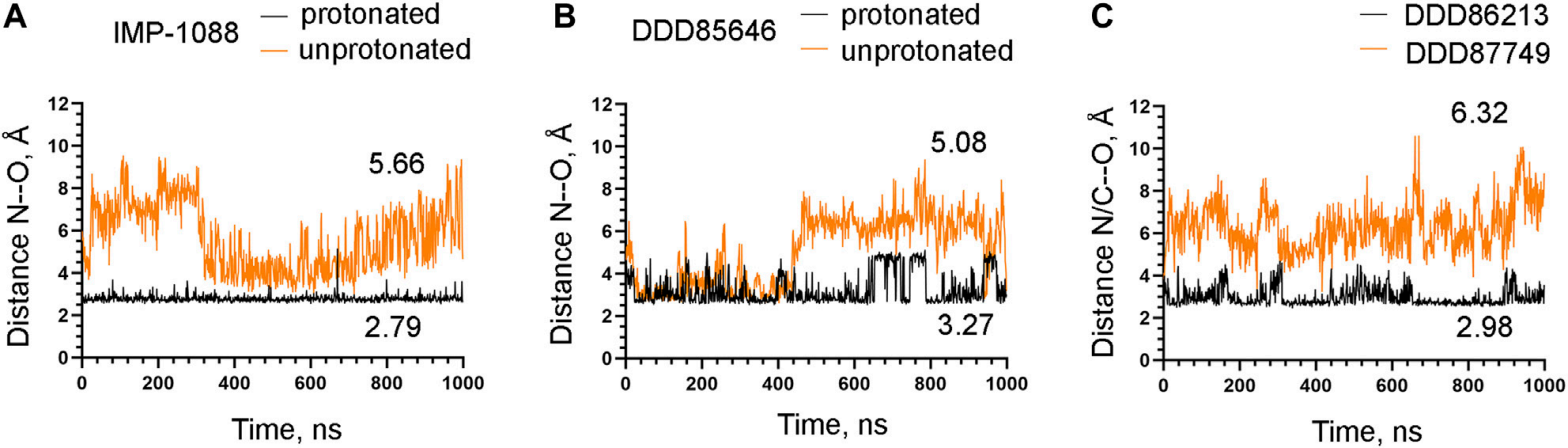
Stability of the interaction between the NMT inhibitors and the C-terminus of the NMT protein. **(A)** The distance between the ligand nitrogen atom and the C-terminal oxygen atom of NMT (N—O distance) in angstroms (Å) during the time course of MD simulations in nanoseconds (ns) of NMT complexes with protonated and unprotonated forms of IMP-1088. **(B)** The distance between the ligand nitrogen atom and the C-terminal oxygen atom of NMT (N—O distance) in angstroms (Å) vs. time of MD simulations in nanoseconds (ns) of NMT complexes with protonated and unprotonated forms of DDD85646. **(C)** The distance between the ligand nitrogen in DDD86213 or the corresponding carbon atom in DDD87749 and the C-terminal oxygen atom of NMT (N/C—O distance) in angstroms (Å) vs. time of MD simulations in nanoseconds (ns) of NMT complexes with DDD87749 and protonated DDD86213. The numbers in the plots indicate the average distance in angstroms (Å). Length < 4Å indicates that the salt bridge is stable; distance > 4Å demonstrates that the interaction between the ligand and the protein is disrupted.

**FIGURE 7:**
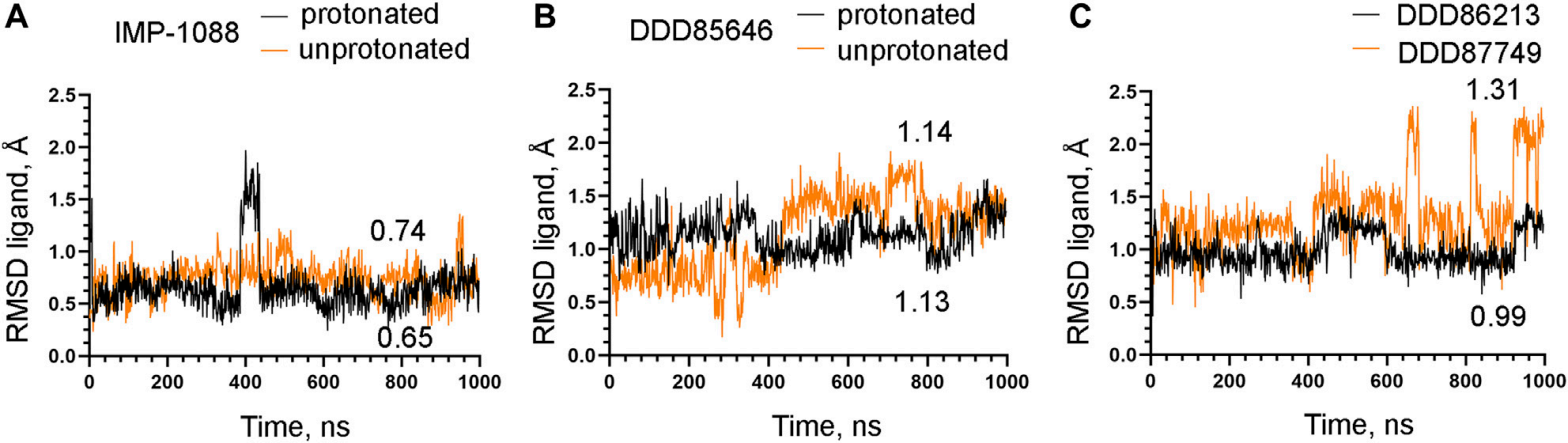
Stability of NMT inhibitor complexes by MD simulations. **(A)** RMSD in angstroms (Å) of the heavy atoms of the protonated and unprotonated IMP-1088 vs. time of MD simulations in nanoseconds (ns). **(B)** RMSD values in angstroms (Å) of the heavy atoms in the protonated and unprotonated DDD85646 forms vs. time of MD simulations in nanoseconds (ns). **(C)** RMSD in angstroms (Å) of the heavy atoms of DDD86213 (protonated form) and DDD87749 vs. time of MD simulations in nanoseconds (ns). The numbers in the plots indicate the average RMSD in angstroms (Å).

To examine the role of the salt bridge in the stability of NMT-ligand complexes, we performed MD simulations using the *Homo sapiens* NMT1 complexes with the unprotonated and protonated forms of IMP-1088 and DDD85646. An excellent representation of the stability of the salt bridge can be obtained by determining the distance between the nitrogen atom of the ligand (acting as a hydrogen bond donor) and the negatively charged oxygen atom at the C-terminus of the NMT protein (acting as hydrogen bond acceptor) (Figures 4E,F), during the time course of MD simulations. The results with the protonated forms of IMP-1088 and DDD85646 indicated that the salt bridge is stable because its length did not exceed the permissible 4 Å distance for a salt bridge length during the entire duration of the MD simulation (Figure 6). Deprotonation of the small molecule eliminates the salt bridge and leads to substantial instability of the interaction between the inhibitors and the C-terminus of NMT (Figure 6). The unprotonated dimethylamino group of IMP-1088 lacks polar hydrogens (Figure 3A); hence, this form is incapable of forming a hydrogen bond with the C-terminus of NMT. This may explain why the unprotonated IMP-1088 forms particularly unstable complexes (Figure 6A). In contrast, the unprotonated piperazine ring of DDD85646 contains a polar hydrogen atom (Figure 3C) that may participate in hydrogen bonding with the C-terminus of NMT. This may explain why the DDD85646 complex with NMT displays relative stability at the beginning to up to 400 ns during the MD simulation (Figure 6B). However, later in the 400-1,000 ns period, the hydrogen bond is disrupted, suggesting that it cannot substitute for the salt bridge (Figure 6B). Similar results were obtained using the NMT complexes with DDD86213 and DDD87749 (Figure 2). For example, the protonated form of DDD86213 formed a stable salt bridge during MD simulations, and the interaction between the inhibitor and the C-terminus of NMT was disrupted due to the replacement of the nitrogen donor atom in DDD87749 with carbon (Figure 6C), As expected, the RMSD values of the heavy atoms for the unprotonated ligand molecules were increased compared to protonated forms (Figure 7). However, the RMSD values of the NMT inhibitors were lower than 1.5 Å, except in part for DDD87749, indicating that the small molecules remained largely restrained to their binding pockets, even though they have lost the interaction with the C-terminus of NMT (Compare Figure 6 and Figure 7). The increased distance between the inhibitor and the C-terminus of NMT in the non-protonated forms compared to protonated (Figure 6) is at least partly due to the conformational movement of the C-terminal region relative to the protein backbone. For example, the average RMSD of the C-terminal amino acid (Gln496) was determined to be 1.10 Å and 3.04 Å in the complexes of protonated and unprotonated IMP-1088 complexes, 2.92 Å and 3.59 Å for the protonated and unprotonated DDD85646 and 1.66 Å and 2.90 Å in DDD86213 and DDD87749 complexes, respectively, indicating that the salt bridge restricts the movement of the C-terminus of NMT.

### 3.4 The salt bridge stabilizes the conformation of the NMT protein

**FIGURE 8:**
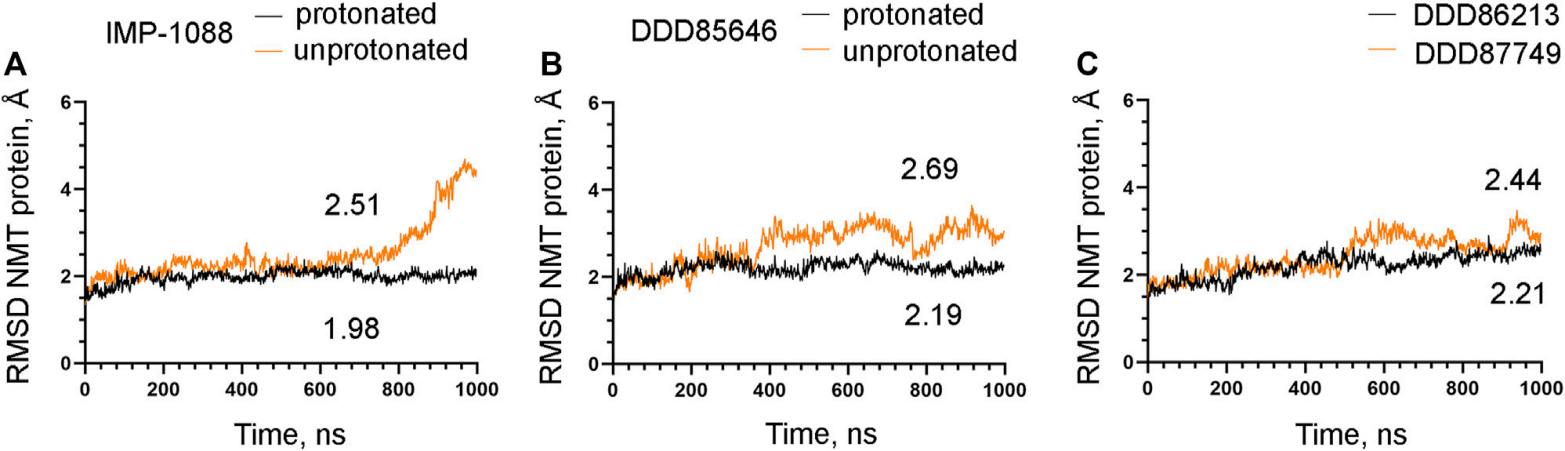
The salt bridge stabilizes the conformation of the NMT protein. The results from MD simulations of *Homo sapiens* NMT1 complexes are shown. **(A)**. RMSD in angstroms (Å) of the heavy atoms of the NMT protein vs. time of MD simulations in nanoseconds (ns) of its complexes with the protonated and unprotonated forms of IMP-1088. **(B)** RMSD in angstroms (Å) of the heavy atoms of the NMT protein vs. time of MD simulations in nanoseconds (ns) of its complexes with the protonated and unprotonated forms of DDD85646. **(C)** RMSD in angstroms (Å) of the heavy atoms of the NMT protein vs. time of MD simulations in nanoseconds (ns) of its complexes with DDD86213 (protonated form) and DDD87749. The numbers in the plots indicate the average RMSD in angstroms (Å).

**FIGURE 9:**
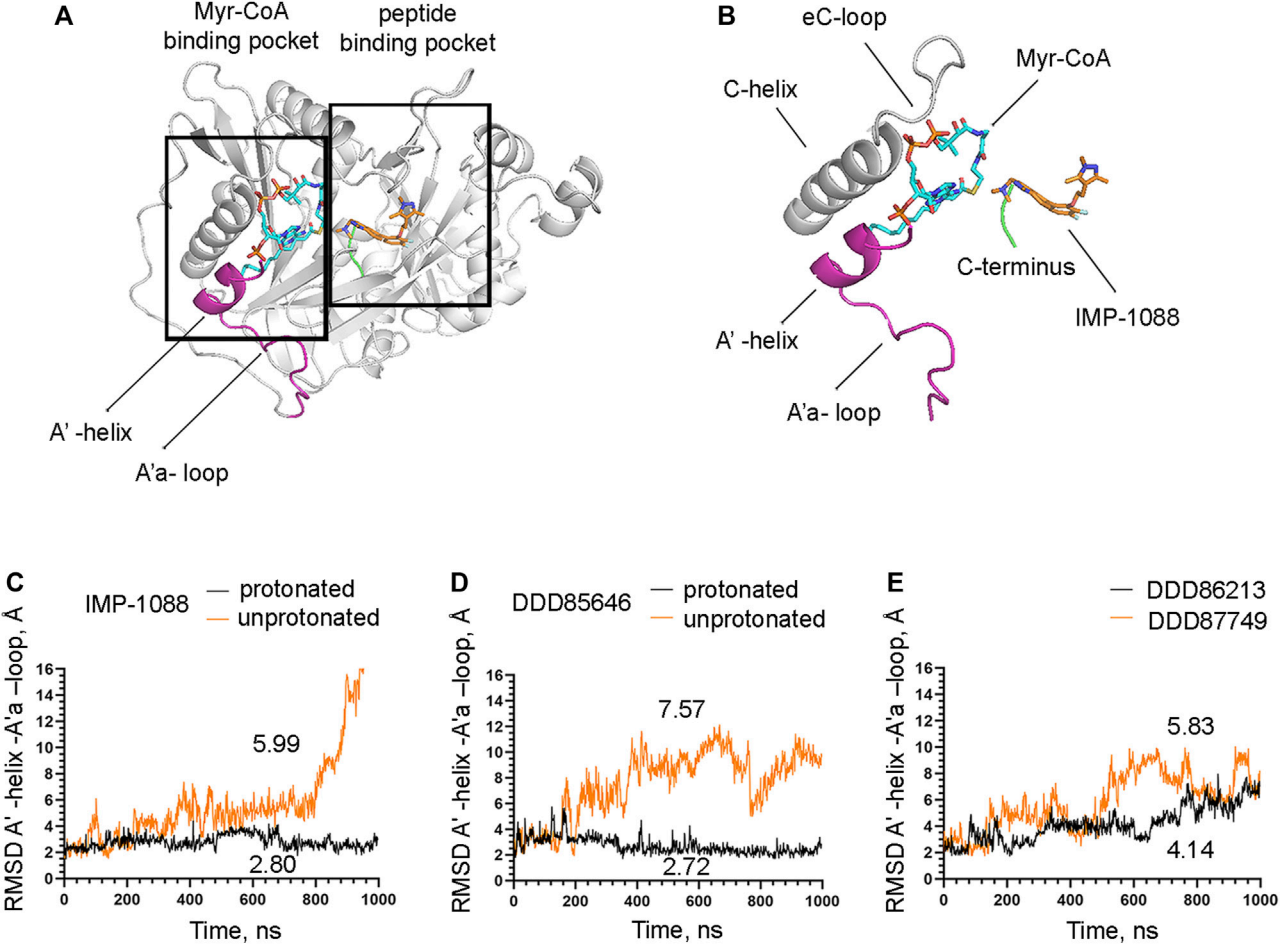
The salt bridge stabilizes the conformation of the Myr-CoA binding pocket. **(A)** The crystal structure of *Homo sapiens* NMT1 (PDB 5MU6), depicting the Myr-CoA and peptide binding pockets **(B)** A zoomed view on the image on the left showing details of the Myr-CoA binding pocket. The A′-helix and A’a-loop, shown in magenta, take part in the formation of the bottom of the pocket **(C)** RMSD in angstroms (Å) of the heavy atoms of the A′-helix and A’a-loop vs. time of MD simulations in nanoseconds (ns) of its complexes with the protonated and unprotonated forms of IMP-1088. **(D)** RMSD in angstroms (Å) of the heavy atoms of the A′-helix and A’a-loop vs. time of MD simulations in nanoseconds (ns) of its complexes with the protonated and unprotonated forms of DDD85646. **(E)** RMSD in angstroms (Å) of the heavy atoms of the A′-helix and A’a-loop vs. time of MD simulations in nanoseconds (ns) of its complexes with DDD86213 (protonated form) and DDD87749. The numbers in the plots indicate the average RMSD in angstroms (Å).

**FIGURE 10:**
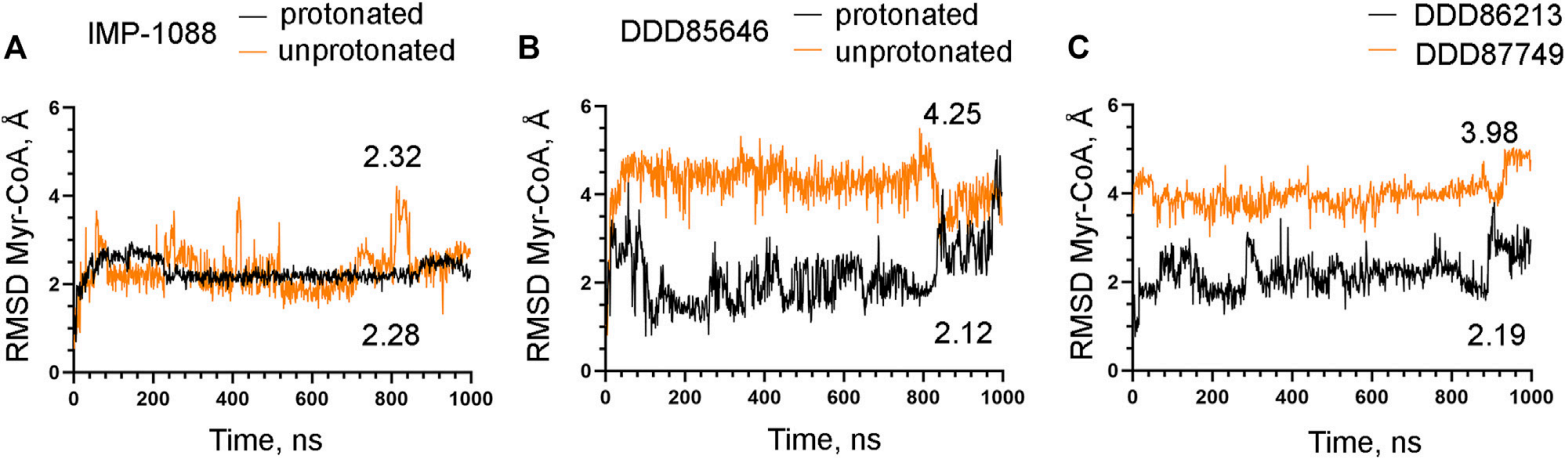
The salt bridge stabilizes the complex between Myr-CoA and NMT. The results from MD simulations of *Homo sapiens* NMT1 complexes are shown. **(A)** RMSD in angstroms (Å) of the heavy atoms of the Myr-CoA vs. time of MD simulations in nanoseconds (ns) of NMT complexes with the protonated and unprotonated forms of IMP-1088. **(B)** RMSD in angstroms (Å) of the heavy atoms of the Myr-CoA vs. time of MD simulations in nanoseconds (ns) of its complexes with the protonated and unprotonated forms of DDD85646. **(C)** RMSD in angstroms (Å) of the heavy atoms of the Myr-CoA vs. time of MD simulations in nanoseconds (ns) of its complexes with DDD86213 (protonated form) and DDD87749. The numbers in the plots indicate the average RMSD in angstroms (Å).

The observation that the NMT inhibitor molecules are restrained into their binding pockets during MD simulations, irrespective of their protonation state (Figure 7), raises questions about how the salt bridge affects ligand potency. Since the salt bridge is an interaction between the inhibitor and the NMT protein, we hypothesize that its effects could also depend on its impact on the protein structure. To test this hypothesis, we compared the dynamic stability of the NMT protein during the MD simulations in complexes with either the protonated or the unprotonated forms of the inhibitors (Figure 8). The RMSD values of the heavy atoms of the NMT protein were significantly increased in the complexes of the unprotonated inhibitors compared to the complexes of the protonated forms (Figure 8), suggesting that the salt bridge stabilizes the conformation of the NMT protein. Similarly, DDD86213, which forms a salt bridge, stabilizes the NMT protein’s conformation compared to DDD87749, which does not (Figure 8). To investigate what specific conformation of the NMT protein was stabilized by the salt bridge, we observed movies of the protein dynamics during MD simulations. Most strikingly, the conformation and the position of the A' -helix and the A’a–loop, which form a part of the Myr-CoA binding pocket (Figures 9A,B), remained stable in the complexes containing protonated IMP-1088, DDD85646 and DDD86213; however, they were dramatically altered in the complexes containing the non-protonated forms of the inhibitors. RMSD calculations confirmed the extraordinary mobility of the A' -helix - A’a–loop region in the complexes with non-protonated inhibitors and its stabilization in the presence of the protonated forms during MD simulations (Figures 9C–E). These results suggest that the salt bridge’s presence stabilizes the conformation of the Myr-CoA binding pocket from which the A' -helix and the A’a–loop are part. Consistent with this, the RMSD values of the Myr-CoA cofactor in the NMT complexes with the protonated inhibitors were reduced compared to those with the non-protonated inhibitors (Figure 10). It was also observed that the eC-loop, located just above the Myr-CoA pocket (Figure 9B), adopts a partially helical conformation in some complexes during MD simulations (not shown). However, this was not related to the inhibitors’ protonation state and is likely not associated with the salt bridge formation.

## 4 Discussion

Here we report that IMP-1088 and DDD85646, two potent NMT inhibitors, form a salt bridge within the active site of the N-myristoyltransferases. The salt bridge is mediated by the interaction between the positively charged groups (dimethylamino group or piperazine ring, respectively) of the inhibitors and the negatively charged carboxyl group at the C-terminus of the enzyme, which is located in the catalytic center of the enzyme. The fact that the salt bridge is formed in the complexes of two structurally unrelated NMT inhibitors, independently identified by high-throughput screening (Frearson et al., 2010; Mousnier et al., 2018; Spassov et al., 2022), implies that the salt bridge may play a special role in NMT inhibition. Indeed, preventing its formation by replacing the nitrogen atom of the ligand, involved in the salt bridge, with a carbon atom dramatically reduces the potency of the inhibitors (Figure 2) (Brand et al., 2017).

Along with the hydrogen, halogen, and chalcogen bonds, the salt bridge belongs to the group of non-covalent interactions, which depend on electrostatic attraction. However, recent findings based on the quantitative Kohn–Sham molecular orbital theory have indicated orbital interactions between the participating atoms, resembling the ones involved in the formation of the covalent bonds, demonstrating that a pure electrostatic model cannot fully describe these non-covalent interactions (Wang et al., 2016; de Azevedo Santos et al., 2021). Since the salt bridge can be viewed as a charge-enforced hydrogen bond, such orbital interactions may also participate during its formation. However, applying the Kohn–Sham molecular orbital theory for the formation of the salt bridge has not been reported in the literature, and the precise molecular orbital interactions during its formation remain to be determined.

Generally, the strength of the salt bridge is highly dependent on the environment (Takano et al., 2000). On the surface of the proteins, the salt bridges may not contribute significantly to interactions, as the gain in free binding energy due to the salt bridge’s formation is insufficient to compensate for the energetic penalty of desolvating its charged groups (Takano et al., 2000; Ferreira de Freitas and Schapira, 2017). In contrast, buried salt bridges can significantly contribute to the binding (Ferreira de Freitas and Schapira, 2017). In this aspect, the salt bridges formed inside the NMT inhibitor complexes are buried into the enzyme’s catalytic center and may possess significant strength.

The salt bridge is the strongest non-covalent interaction and participates in protein folding and protein-protein interactions, and it is known to contribute to protein conformational stability as well (Kumar and Nussinov, 2002; Bosshard et al., 2004; Donald et al., 2011; Basu and Mukharjee, 2017; Ferreira de Freitas and Schapira, 2017). However, the role of salt bridges in drug-protein interactions has remained not well understood. In a recent study, Kurczab et al. identified 1122 unique small molecule ligands from the Protein Databank that form salt bridges with their protein targets, indicating that salt bridges are frequent in drug-protein interactions (Kurczab et al., 2018). The set contains structures from different enzyme classes, including hydrolase, transferases, reductase, oxidoreductase, lyases, and certain G protein-coupled receptors (GPCRs) (Kurczab et al., 2018). In several cases, it has been experimentally shown that the salt bridge plays a critical role. For example, the Epidermal Growth Factor Receptor (EGFR) inhibitor, compound 10, forms a salt bridge with Asp831 in the kinase domain of EGFR through its terminal dimethylamino group (Peng et al., 2013; Ferreira de Freitas and Schapira, 2017). Replacing the nitrogen atom of this group with a carbon atom reduced the potency of this compound by over 800 folds (from IC_50_ 29 nM to IC_50_ 25 µM) (Peng et al., 2013; Ferreira de Freitas and Schapira, 2017). In addition, it has been reported that preventing the formation of the salt bridge between the aminergic ligands and the aminergic class A GPCRs by mutating the Asp3.32 residue, which is engaged in the salt bridge, to Ala reduces the binding 126-fold (Kurczab et al., 2018). In comparison, the absence of the salt bridge reduces the activity of the NMT inhibitors by over 1,300 fold (Figure 2) (Brand et al., 2017). Although the salt bridge is stronger than the hydrogen bond, such substantial differences in potency in the pairs of compounds that are capable of forming salt bridges or not are difficult to explain by a simple increase in the strength of the interaction between the ligand and the protein and may suggest the existence of a more complex mechanism that mediate or at least amplify the salt bridge’s effects.

The mechanisms that mediate the salt bridge effect on the inhibitors’ potency were investigated by MD simulations using NMT complexes with the protonated and unprotonated forms of the inhibitors. These forms of the inhibitors are best suited to study the role of the charge of the molecule and the salt bridge with minimum structural change. In DDD85646, this allows comparing compounds that form a hydrogen bond (unprotonated DDD85646) to compounds that form a salt bridge (protonated DDD85646). However, considering the pKa of the IMP-1088 and DDD85646, the unprotonated forms may rarely exist under the physiological pH, and thus the interaction between unprotonated forms and NMT may not occur under these conditions. To address this point, we also performed MD simulations of NMT complexes with DDD86213 and DDD87749, where the activity of the compounds under physiological pH has been determined (Figure 2). The results demonstrated the significance of adding a charge species to the DDD87749 scaffold and how this can be used to increase the ligands’ potency. The inability of the inhibitors’ non-protonated forms to participate in a salt bridge leads to the instability of their association with the C-terminus of NMT (Figure 6). This does not lead to the dissociation of the inhibitor molecules, at least in the 1 µs time frame of the MD simulations, as they remain largely confined to their binding pockets (Figure 7). However, the salt bridge’s presence stabilizes the NMT protein’s conformation (Figure 8). Interestingly, the activity of the compounds IMP-1088 > DDD85646 > DDD86213 > DDD87749 corresponds to the compounds’ ability to stabilize the NMT protein’s conformation (Figure 8), implying that the stabilization of NMT conformation by the salt bridge may determine their potency. Among the protonated forms, DDD86213 has the weakest capacity to stabilize the NMT conformation (Figure 8), consistent with its lower inhibitory activity and that DDD86123 is an intermediate lead compound in the DDD85646 series and its activity has not been wholly optimized (Figure 2).

The salt bridge’s effect on the protein conformation is particularly evident in the A′- helix and the A’a-loop, which forms part of the Myr-CoA binding pocket (Figure 9). By stabilizing the conformation of the Myr-CoA binding pocket, the salt bridge exerts a positive effect on the stability of the complex between NMT and its cofactor Myr-CoA (Figure 10). By doing so, the salt bridge may also indirectly exert a positive effect on the stability of the whole complex. For example, it has been reported that the binding affinity of DDD85646 to NMT is increased 33-fold in the presence of Myr-CoA (Frearson et al., 2010); hence the stability of the NMT-Myr-CoA complex may translate into increased stability of the interaction between the NMT inhibitors themselves and the NMT protein. In addition to the more pronounced conformational changes in the Myr-CoA binding pocket, smaller conformational changes exist in other protein regions, including the C-terminus of NMT. Thus the stabilization of regions of the protein structure by the salt bridge, other than the Myr-CoA binding pocket, may also play a role in the compounds’ activity.

Salt bridges have been reported to function as molecular clips that stitch together large surface areas at interacting protein interfaces (Basu and Mukharjee, 2017). Similarly, the results reported here suggest that the formation of a salt bridge between the protein receptor and the ligand may stitch a particular protein region and by restricting its conformational movements to exert a stabilizing effect on the overall protein conformation. Therefore, the salt bridge’s impact on the inhibitors’ potency could be more complex than previously anticipated and may involve, at least in part, the stabilization of the protein receptor conformation. This finding may not be surprising in the light that salt bridges, in general, are known to contribute to protein conformational stability (Takano et al., 2000; Bosshard et al., 2004).

In the current work, the parameters for MD simulations were optimized for studying protein conformational dynamics. The systems were simulated with the ff14SB force field (Hornak et al., 2006; Maier et al., 2015; Case et al., 2018), specially parameterized and recommended for protein dynamics as a part of the AMBER v. 18 package (Case et al., 2005; Case et al., 2018). ff14SB (Maier et al., 2015) has evolved from the ff99SB force field and includes improvements in the torsional parameters for the backbone and side chains (Hornak et al., 2006; Case et al., 2018). Frames were saved every 1 ns to generate 1,000 frames for a total of 1 µs duration of MD production simulations to detect significant events in the nanosecond time scale. These include protein conformational changes such as side chain rotations, backbone and loop motions, and ligand binding. Statistical inefficiency, principal component analysis, and wavelet analysis could be used to identify significant events during MD simulations to reduce the number of frames of MD simulations without losing statistical power (Heidari et al., 2016; Gonçalves et al., 2017). However, considering the number of frames analyzed in the nanosecond time range in the current study, most, if not all, of the significant events related to protein conformational changes and ligand binding are expected to be captured during analysis.

During the MD simulations, some conformational changes in the NMT protein were observed that were not related to the protonation state of the inhibitors and hence are not dependent on the presence or the absence of the salt bridge; nevertheless, they might be of interest to researchers studying the N-myristoyltransferases. Specifically, it was observed that the eC-loop, positioned just on top of the Myr-CoA binding pocket, opens and adopts a partially helical composition in some MD simulations. An analogous loop, the Ab-loop, is located on top of the adjacent peptide-binding pocket (Dian et al., 2020). The Ab-loop has been reported to exist in open and closed conformations and controls the entry of the substrate peptide into the catalytic center (Dian et al., 2020). The observation of alternative conformations of the eC-loop suggests the possibility that this loop may also perform a similar role in regulating the entry of Myr-CoA into its binding pocket. However, this remains to be determined in future studies.

The salt bridges typically involve the amino acid side chains of Asp or Glu if the ligand is positively charged or the side chains of Lys or Arg if the ligand is negatively charged (Ferreira de Freitas and Schapira, 2017; Kurczab et al., 2018). The occurrence of a salt bridge with the carboxyl group at the C-terminus of the protein is probably unique to N-myristoyltransferases and possibly is a reflection of its unusual localization in the active site of the enzyme and its catalytic functions (Dian et al., 2020). According to Uniprot (UniProtConsortium, 2021), the C-terminal amino acid residue in *Homo sapiens* NMT1 and NMT2 is Gln. Gln is also the C-terminal residue in NMTs of the vertebrate species, including *Danio rerio*, *Xenopus laevis*, *Gallus gallus*, and *Mus musculus*. In the protozoan species, the identity of the C-terminal amino acid is not conserved—it is Val446 in *Trypanosoma brucei* NMT, Leu410, and Leu421 in *Plasmodium falciparum* and *Leishmania major* NMTs. This may not be surprising considering that the side chain of the C-terminal amino acid is orientated in the opposite direction of the active site and is not involved in the catalysis (Dian et al., 2020). However, the conservation of the C-terminal residue of NMT in all vertebrate species may suggest that the side chain of this residue may play a role that remains to be determined.

The positive charge of the NMT inhibitors may influence other pharmacological properties of theirs, such as cell membrane or BBB permeability, absorption, and distribution. It is estimated that about 40% of approved prescription drugs are positively charged and exist as organic cations at neutral pH (Uddin et al., 2021). The transport of such organic cations across the membrane could be dependent on or facilitated by uptake from transport carrier proteins (Thomas et al., 2004; Sprowl et al., 2016). Irrespective of the entry mechanism, the NMT inhibitors, as documented in numerous articles, are undoubtedly capable of crossing the cell membrane and can effectively target the N-myristoyltransferases intracellularly both in cell culture models and *in vivo* (Thinon et al., 2014; Beauchamp et al., 2020).

In conclusion, the results suggest that salt bridges could be used as valuable tools in drug design. The inclusion of charged chemical groups in the ligand structures, where they can participate in salt bridges with the target proteins, analogous to the case of DDD87749 and DDD86213, could lead to a significant increase in activity. Moreover, the results also imply that conformational stabilization of the target protein structure could be a hallmark of the salt bridges’ effects. In this aspect, studying the protein dynamics by MD simulations could be used to predict the outcome of introducing a new salt bridge in a specific protein-ligand complex.

## Data Availability

The original contributions presented in the study are included in the article, further inquiries can be directed to the corresponding author.
